# Music Influences Hedonic and Taste Ratings in Beer

**DOI:** 10.3389/fpsyg.2016.00636

**Published:** 2016-05-06

**Authors:** Felipe Reinoso Carvalho, Carlos Velasco, Raymond van Ee, Yves Leboeuf, Charles Spence

**Affiliations:** ^1^Department of Electronics and Informatics, Vrije Universiteit BrusselBrussels, Belgium; ^2^Department of Experimental Psychology, KU LeuvenLeuven, Belgium; ^3^Crossmodal Research Laboratory, Department of Experimental Psychology, University of OxfordOxford, UK; ^4^Philips Research Laboratories, Department of Brain, Body and BehaviorEindhoven, The Netherlands; ^5^Department of Biophysics, Donders Institute, Radboud UniversityNijmegen, Netherlands; ^6^The Brussels Beer ProjectBrussels, Belgium

**Keywords:** taste, sound, music, perception, experience design, gastrophysics

## Abstract

The research presented here focuses on the influence of background music on the beer-tasting experience. An experiment is reported in which different groups of customers tasted a beer under three different conditions (*N* = 231). The control group was presented with an unlabeled beer, the second group with a labeled beer, and the third group with a labeled beer together with a customized sonic cue (a short clip from an existing song). In general, the beer-tasting experience was rated as more enjoyable with music than when the tasting was conducted in silence. In particular, those who were familiar with the band that had composed the song, liked the beer more after having tasted it while listening to the song, than those who knew the band, but only saw the label while tasting. These results support the idea that customized sound-tasting experiences can complement the process of developing novel beverage (and presumably also food) events. We suggest that involving musicians and researchers alongside brewers in the process of beer development, offers an interesting model for future development. Finally, we discuss the role of attention in sound-tasting experiences, and the importance that a positive hedonic reaction toward a song can have for the ensuing tasting experience.

## Introduction

There is growing evidence to support the claim that multisensory information can be used to improve the design of food/beverage products, as well as the design of dining experiences (see Spence, 2015b, for a review). For instance, by systematically manipulating the different sensory cues that are involved in the process of eating and drinking, it is possible to positively impact the overall eating and drinking experience (see Spence and Piqueras-Fiszman, 2014, for a review). Exploring our senses and the way in which they interact while eating and/or drinking, has opened up the way for novel trends, such as “Sensploration”, and concepts, such as “Gastrophysics.” These trends and concepts are contemporary with the interests of upcoming generations that are undoubtedly seeking out experiences that will “promote their senses” (Spence and Piqueras-Fiszman, 2014; Leow, 2015).

In particular, research shows that what we see and hear can exert a significant influence over our perception of, and hedonic responses to, flavors (Spence et al., 2015; see Favre and November, 1979; Seo and Hummel, 2015; Spence, 2015a, for reviews). So, for example, researchers have documented that the curvature of different design elements on a product's packaging can influence the expected taste of a product, with people expecting sweeter tastes for rounder designs and sourer tastes with more angular designs (Lunardo and Livat, 2016; see also Velasco et al., 2016, for a review). Researchers have also documented that the shape of the food, and even the shape of a plate on which it is served, can influence the perception of taste (e.g., sweetness; see Fairhurst et al., 2015; see Spence and Piqueras-Fiszman, 2014, for a review). Furthermore, the different colors that may be used on product packaging/labeling have also been shown to lead to different flavor associations (e.g., Piqueras-Fiszman and Spence, 2011), and to influence the way in which people search for flavor information (for example, when the color information is congruent vs. incongruent with the associated flavor, Velasco et al., 2015). That said, recent reports have evaluated whether sensory interventions can add value by influencing, for example, a customer's willingness to pay. For instance, Michel et al. (2014) demonstrated that consumers are willing to pay significantly more for art-inspired plating, as compared to neat and simple ways of arranging the food on the plate. Notably, such plating techniques already caught the attention of major retailers, such as Lidl^1^.

It is important to point out that the way in which foods and drinks are perceived prior to tasting is mostly related to visual and orthonasal sensory inputs (Spence and Piqueras-Fiszman, 2014). Nevertheless, a spate of recent studies has also highlighted the significant influence that sound can have on how we perceive foods and drinks, considering that this can add significant value (not to mention pleasure) to the consumer's overall eating/drinking experience (e.g., Reinoso Carvalho et al., 2013, 2015c; see Spence and Shankar, 2010; Spence, 2015a, 2016, for reviews). For instance, Reinoso Carvalho et al. (2015a, 2016, in press) and Crisinel et al. (2012) have demonstrated that by following the literature (as a baseline for the production of sonic stimuli), it is possible to compose music and soundscapes that systematically, and specifically, modulate the perceived flavor of food and/or drinks.

Reinoso Carvalho et al. (2015a) conducted a study in which three soundtracks were produced, one designed to be congruent with sweetness, another with bitterness, and the third somewhere in-between (see Spence and Shankar, 2010; Knoeferle and Spence, 2012, for overviews). The results revealed that what people hear exerts a significant influence over their ratings of the taste of the chocolate. Moreover, Reinoso Carvalho et al. (2015b) recently demonstrated that customers are willing to pay significantly more for a chocolate that comes with its own song, than when no song comes with it. Another study that looked for crossmodal correspondences between classical music and wine showed that people perceived a wine as tasting sweeter, and reported enjoying the experience more while listening to matching music, than while tasting the wine in silence (Spence et al., 2013; see also Spence et al., 2014). Moreover, in an early study designed to assess the influence of sonic cues on consumer behavior, Areni and Kim (1993) reported that customers were willing to spend significantly more for a bottle of wine when classical music was played in the background, as compared to “Top-40” pop music.

The experiment presented here was designed to assess the way in which background music and packaging/labeling would influence people's perception of the taste of a beer. Most of the time, of course, food/beverage products come labeled, but they do not, at least not yet, usually come with a customized soundscape presented as part of the eating/drinking experience (this representing an interesting opportunity for brand managers and marketers). Therefore, from a design perspective, here we hypothesize that people may easily feel compelled to focus on the novelty of a sound-tasting experience. Likewise, loud background music can lead to increased alcohol consumption (Guéguen et al., 2008). Furthermore, Drews et al. (1992) reported that the presence of music in bars tends to increase the length of stay and amount of beer that is consumed. Though, in their experiment, the drinking rate was unaffected by the music. Visual and sonic cues can also be used to bias consumer drinks choices, when included as part of the restaurant scene (Sester et al., 2013; cf. Wansink and Van Ittersum, 2012).

The purpose of the present experiment was to assess whether the process of brewing and presenting a beer could be enriched by means of related visual and auditory information. In order to differentiate our assessment from previous studies in the area, we wanted to consider the fact that most drinks come in some kind of packaging. Therefore, we also manipulated the presence vs. absence of labeling, in order to assess the potential effect of the beer's label, and its interaction with music, on the tasting experience. Finally, as part of this exercise, we envisioned the multisensory customization of beer experiences as a way to offer enhanced enjoyment (and perhaps also increase brand loyalty).

In the experiment reported here, different groups of customers experienced a beer under three different conditions. The first serving as a baseline. The second condition assessed the influence of the bottle's label. The third condition added a customized piece of music to the tasting experience. Note that those previous studies that have assessed the influence of visual cues on the tasting experience have all tended to manipulate one or two visual features, whereas we were interested in assessing the influence of the label (i.e., a more complex combination of visual elements, one that had higher ecological validity), as a whole, and comparing it with the effect of the song. The beer used for this experiment was the product of a collaboration between a brewery—The Brussels Beer Project (TBP, Belgium), and a music band/group. It is this process of co-creation between artists, researchers, and designers that will be analyzed here. That being said, from a perceptual standpoint, we also hypothesized that the visual and sonic designs that inspired the creation of the formula for the beer might influence the way in which the beer was perceived. In particular, we expected a plausible interaction between the beer's visual identity, music, and taste, based on the proposed interdisciplinary design. Here, we hypothesized that the extent to which people liked the beer would be affected by the visual and auditory information presented together with the beer (think of congruence in terms of designer intuitions that could lead to fluency and liking, Velasco et al., 2016). Moreover, given the spectral analysis of the music, we also expected that the song would influence the sour and sweet ratings of the beer.

## Materials and methods

### Participants

Two hundred and thirty-one participants [163 males, 68 females, Mean (*M*) age = 35.80 years, standard deviation (*SD*) = 10.90—all of the participants were, at least, 18 years of age] took part in the study after giving their informed consent. None of the participants reported having a cold or any other sensory impairment (smell, taste, or hearing) at the time of the study. Sixty-one percent of the participants responded to the survey in English and 39% in French. Eighty-three percent of the participants declared knowing TBP. Summarizing, the great majority of the participants were TBP clients from Belgium and its surroundings^2^.

### Stimuli

**Figure 1:**
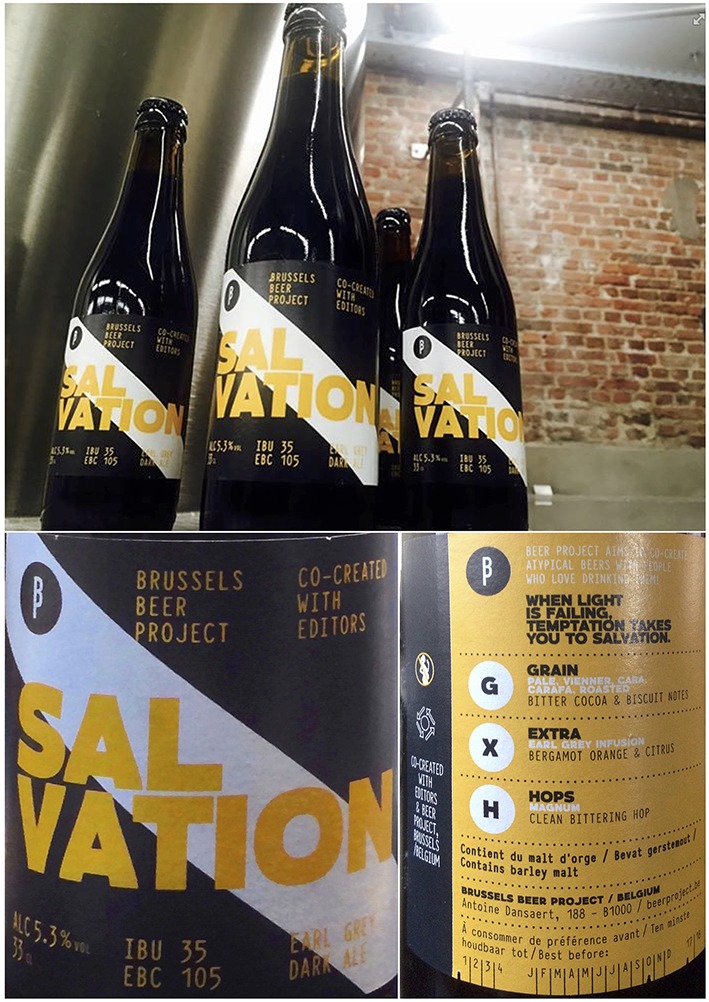
**Visual presentation of the “Salvation” beer: full bottle (top), zoom-in on front (bottom-left), and back label (bottom-right)**. Dimensions of the front label (7 × 9 cm) and the back label (7 × 6 cm)—height × width. The bottle is a template 33cl Belgian beer bottle, commonly used among most small-artisanal Belgian brewers.

The beer used in this experiment, a limited edition named “Salvation,” is a co-creation between TBP and an UK music band called “The Editors^3^” (TE). The complete description of the creative process involving the development—and characterization—of the experimental taste and sonic stimuli can be accessed at the following link: http://tbpeditors-experience.tumblr.com/; Retrieved on January 2016). The bottle, front and back labels are shown in Figure 1.

#### Beers

**Figure 2:**
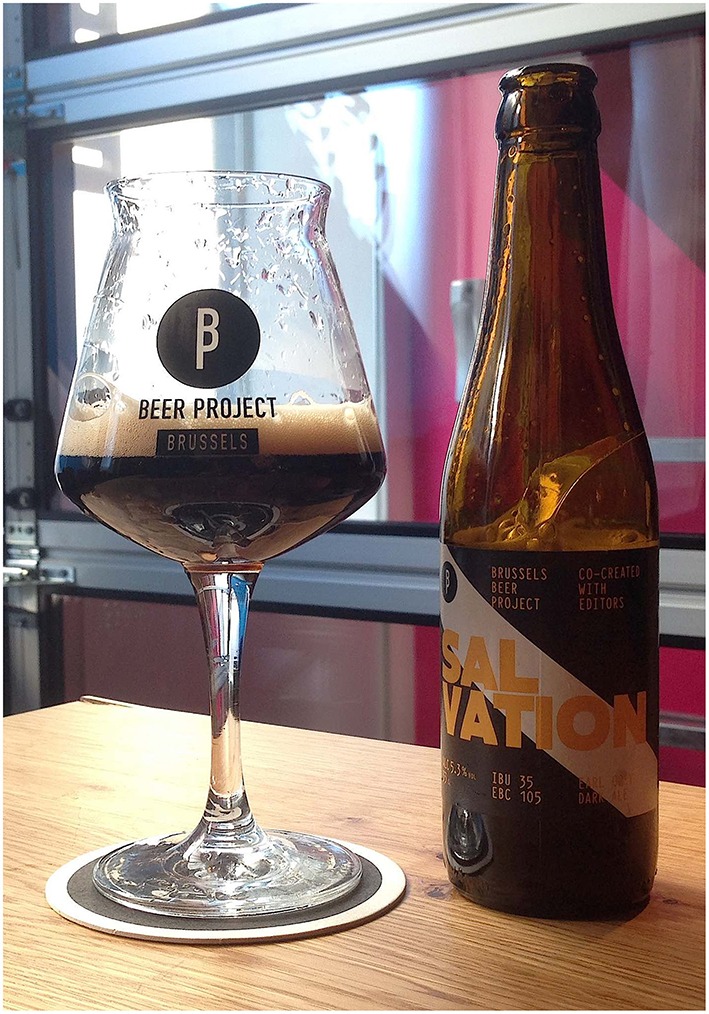
**Beer stimulus as it was presented to the participants in Conditions B and C**. In Condition A, this presentation didn't include any labeling on the bottle.

The formula of the beer is based on a UK porter style. Porter beers constitute a sub-category of general dark ale beers. They are considered as dark beers in the EBC color scale^4^ and have medium body. As an element of the association with the UK, an Earl Grey infusion was added to the beer's formula. Earl Grey tea is aromatized with bergamot orange, giving it a distinctive citrus note in the flavor. The formula is also composed by the following grains: pale, vienner, cara, carafa, and roasted barleys. This combination of malts gives bitter cocoa and biscuit flavor notes. Finally, a classic-hop is also included, with moderated bittering value (IBU)^5^, in order to add a baseline of bitterness to the final formula. Its alcohol strength is labeled as 5.3%. Figure 2 shows a picture of the beer, as it was served in the experiment. Note that the glass used in this experiment is a TBP glass. In Belgium, each brewery (and sometimes, each type of beer) has its own glass and is generally served in such matching glassware (cf. Spence and Wan, 2015).

#### Visual stimuli

A customized label was produced for this beer. The label was conceived as a blend between TBP's current label templates, and TE's visual identity. The name of the beer, TBP logos, and indications of bitterness (IBU), color (EBC), and alcohol content appears on the front label. The back label mentions the varieties of hops, yeast, and malt used in its formulation. TBP chose Stout Capital as the font to use in this label^6^. The label colors follow those used on TE's latest album cover, namely “In Dream” (black, white, and yellow)^7^. They used black as the main color for the front label (as that was the most dominant color in the mentioned cover), the characteristic diagonal of TBP logos in white^8^, and yellow as complimentary “rays of lights” (see Figure 1).

#### Auditory stimuli

**Figure 3:**
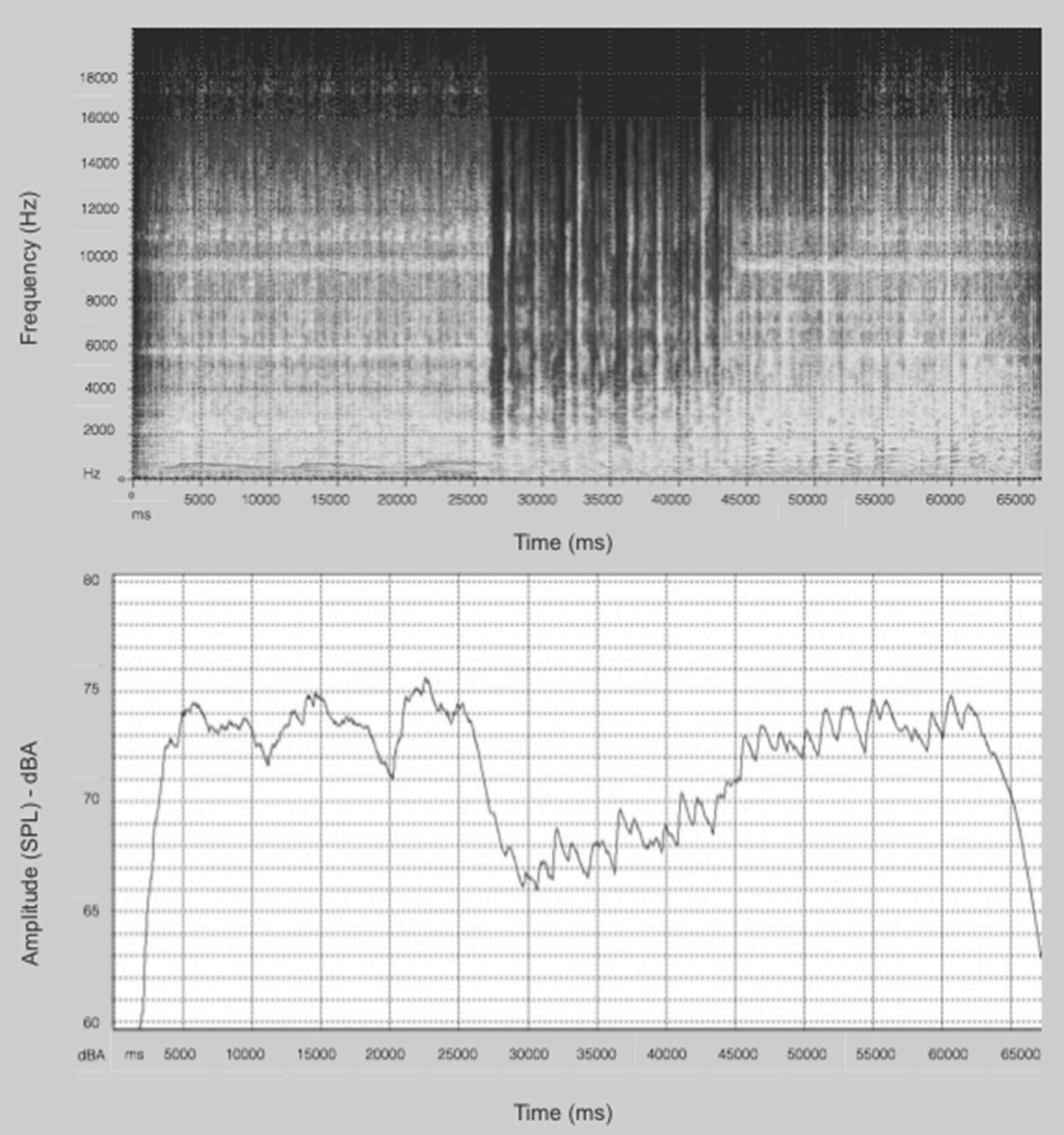
**Frequency vs. Time vs. Amplitude Spectra (top), and Amplitude vs. Time Spectra (bottom) of the song's fragment (Figure source: dBSONIC)**.

A fragment of the song “Oceans of Light,” from the previously-mentioned album was chosen for use in this experiment. The fragment contained around 1 min of the original song (from min 2:25 to min 3:25, approximately^9^). Figure 3 shows the spectral and temporal features of the song. By relating the musical and psychoacoustic analysis with the summary of the crossmodal correspondences between basic tastes and sonic elements presented by Knoeferle and Spence (2012), the suggestion would be that the song might enhance the perceived sourness of the beer. For example, in Knoeferle and Spence's Table 1, which summarizes the results of a number of studies carried out by different research groups, high spectral balance, staccato articulation, syncopated rhythm, high pitch, among others, are musical/psychoacoustic elements that correspond to sourness. Furthermore, due to the predominant piano in the second verse, the song might also be expected to have an effect on the perceived levels of sweetness (Knoeferle and Spence, 2012). Following the aforementioned literature, no predominant musical/psychoacoustic elements that might be expected to have a modulatory effect on the perceived bitterness were detected.

**Table 1:** ***Mean*s and *SD*s of the sound-taste ratings in Condition C**.

**Question**	***Mean***	***SD***
1. How interested are you in music?	5.41	1.33
2. How much do you like this song?	5.04	1.43
3. How much do you think this song matches with the beer's taste?	4.70	1.30

*Most of the participants liked the song and agreed that it matched the taste of the beer (ratings based on 7-point Likert scale, with 1 being “Not at all,” and 7 “Very much”)*.

### Design and procedure

#### Design

The experimental protocol was approved by the Social and Societal Ethics Committee at KU Leuven (Protocol: G-2015 09 337). The experiment was subdivided into three main stages. In the first, the participants had to input their personal details, and read and accept the terms of the informed consent in order to proceed. The second and third stages were different for each of the three experimental conditions/days (different participants took part in each of the three experimental conditions/days). In Condition A (day 1), the participants evaluated the beer presentation without any label on the bottle, tasted the beer afterwards and rated their beer-tasting experience (by means of hedonic ratings, taste attributes and alcohol strength). Here, they did not have any information as to the origin of the beer. Note that when designing this experiment, it was important to consider that the assessment of how sound can influence taste does not necessarily come easily to naive participants. That said, an effective way to focus their attention on the expected multisensory cues was necessary as part of the experimental design (think of background noise, people coming and going etc., as disturbance that could have an impact on the concentration levels of the participants). Therefore, for Conditions B and C, a different written message was delivered to the participants. In Condition B (day 2), the participants evaluated the beer presented with its label on the bottle, tasted the beer afterwards, and rated their beer-tasting experience. Here, they were informed that the beer that they were tasting resulted from a collaboration between TBP and The Editors (band). Finally, in Condition C (day 3), the participants evaluated the beer's presentation with its corresponding label, tasted the beer while listening to the chosen song, and rated their beer-tasting experience. The participants in this condition were told that the beer resulted from a collaboration between TBP and The Editors (band), and that the song that they listened to was the source of inspiration for the formulation of this beer. The questions used in steps two and three were fully randomized.

The survey that the participants used to rate their experience consisted of an electronic form, containing multiple choices, 7-point Likert-rating-scales (mostly with 1 being “Not at All” and 7 “Very much”), and YES–NO questions. As main tasks, all of the participants had to rate how much they liked the beer, and their willingness-to-pay, before and after tasting. They were also asked to rate three basic taste components of the beer while tasting it (sweetness, bitterness, and sourness), and its perceived alcohol strength.

#### Procedure

A private invitation was sent to TBP crowd-funders to join this experiment (that took place between the 12^th^ and 14^th^ of November, 2015). They were invited to be part of a scientific experiment involving beer tasting and the senses. Those funders who accepted the invitation, and those customers who visited the brewery between 5 and 9 p.m., were invited to take part in this study.

TBP's brewery is subdivided in three main areas (bar at the entrance, bar in the back, and brewing area). For our set-up, a private table was set-up in the back of the brewing area. The full set-up allowed for 10 participants to join the experiment at once. Since the experiment was performed during evening hours, the artificial light in the experimental area was kept to a minimum, in order to provide a more “intimate” ambience. The lighting levels were also adjusted in order to provide enough light for the necessary reading and visual evaluations of the experience.

**Figure 4:**
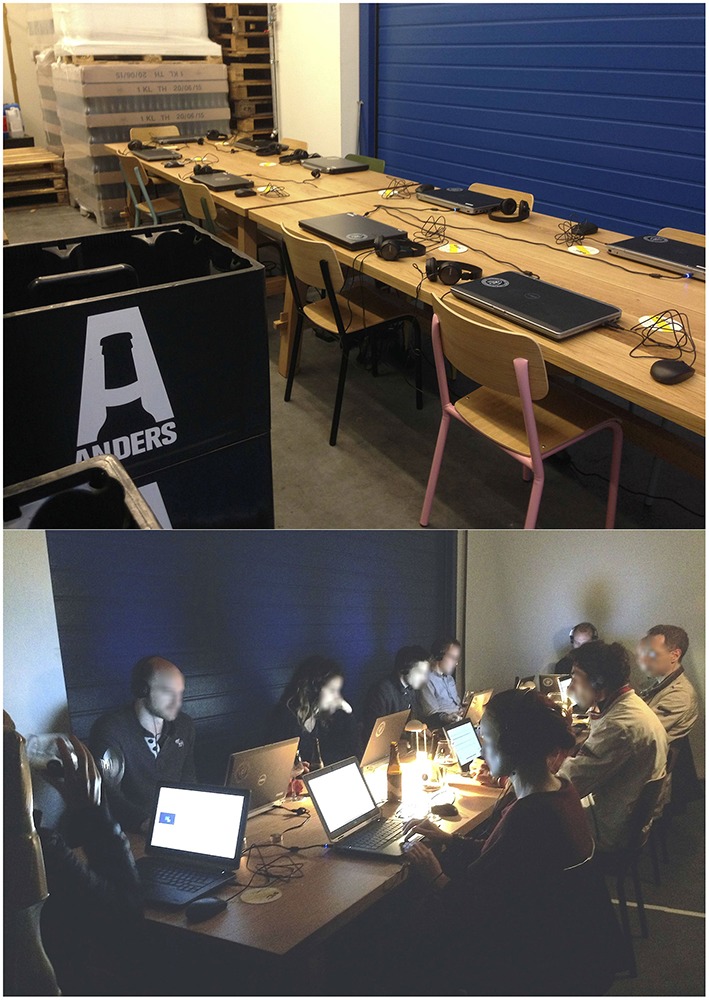
**Experimental area before (top), and during (bottom) the experiment**.

When taking part in the experiment, each participant was seated in front of a computer screen. Each participant had a pair of headphones, a computer mouse, and a keyboard to interact with the survey. Each sound reproduction system was set to 35% of sound power and double checked in order to ensure that each participant was exposed to the soundtracks at approximately the same sound pressure level (L_eq1m_ = 70 ± 2.5 dB). The soundtrack was presented over SONY MDRZX310 headphones. Since the experimental area was far away from both bar zones, the background noise during the experimental hours was kept fairly low. Nevertheless, in order to improve their attention—and with the objective of eliminating background noise as a factor, since the background noise conditions changed throughout the days and timeframes—all of the participants wore headphones during the experiment, even though no sonic stimulus was presented in Conditions A and B. Furthermore, all the brewing machines were off during experimental hours, therefore no industrial noise was present. Figure 4 shows the configuration of the experimental area.

While in the experiment, the participants were informed that they would be given complimentary beer to taste while sometimes listening to sound, and answering a survey. A full bottle of 33 cl was provided to each participant, but only a fixed dose of ~10 cl of beer was served during the experiment.

One supervisor was present during the entire process to provide guidance, coordination, and support, together with the written guidelines concerning the experiment. Upon finishing, the participants were instructed to leave the experimental area without discussing any details with the next group. Tap water for mouth washing was available before, during, and after the experimental procedure for anyone who requested it.

## Results

From the 231 participants, 73 participated in Condition A, 79 in Condition B, and 79 in Condition C. Outliers were removed from the data set (<5% of data points removed; note that the results followed the same pattern when the outliers were included). The Bonferroni correction was applied to all *post-hoc* tests for multiple comparisons that are presented in this study.

### Evaluating the hedonic aspects of the tasting experience

Before tasting, the participants rated how tasty they expected that the beer would be. After tasting, the participants rated how much they liked the beer's taste. A 3 × 2 mixed design analysis of variance (ANOVA) with condition (A, B, and C) as the between-participants factor, and time (before vs. after tasting) as the within-participants factor, was performed. The participants liked the beer just as much before as after tasting; the average mean before tasting was 4.76 (*SD* = 1.16), and after tasting was 4.80 (*SD* = 1.13), *F*_(1, 216)_ = 0.169, *p* = 0.682, η^2^_*p*_ = 0.001. No significant differences were obtained between conditions, *F*_(2, 216)_ = 0.380, *p* = 0.684, η^2^_*p*_ = 0.004. Moreover, no interaction between before-after ratings and conditions, *F*_(2, 216)_ = 2.576, *p* = 0.078, η^2^_*p*_ = 0.023, was found, though a trend was present.

**Figure 5:**
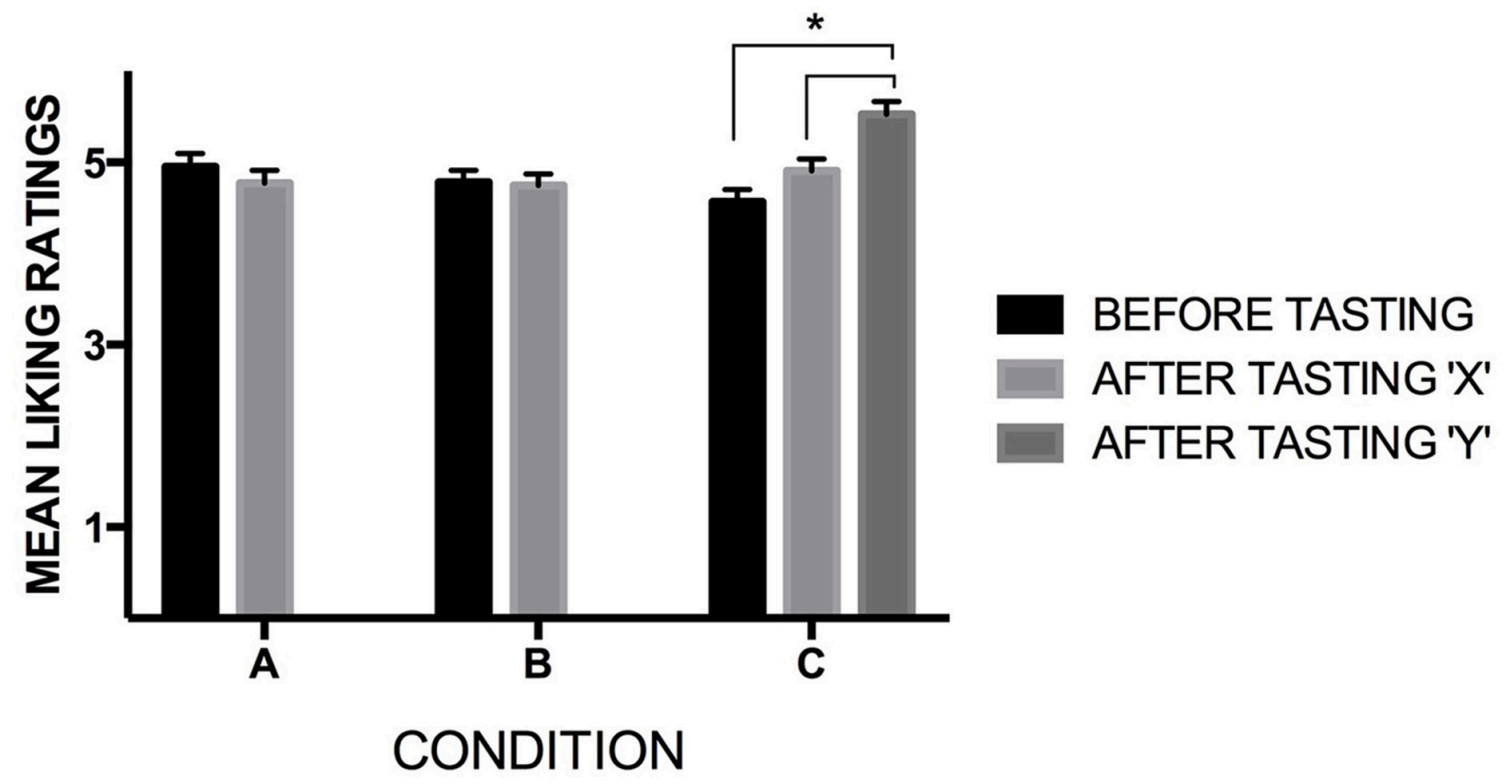
**Mean ratings of the evaluation of the subjective aspects of the tasting experience, with “X” being the ratings of how much they liked the beer (X), and “Y” the likeness ratings of the sound-tasting experience (Y) (ratings based on 7-point scales, being 1 “not at all,” and 7 “Very much”)**. Visualizing these evaluations, it seems that the participants valued the customized soundscape component of the multisensory beer-tasting experience. The error bars represent the standard error (*SE*) of the means here and in all the other graphs of the present study. Significant differences between the specific interactions are indicated with an asterisk “*” [*p*-value for the comparison before-tasting and after-tasting ratings “Y” (*p* = 0.001); *p*-value for the comparison after-tasting ratings “X” and “Y” (*p* < 0.001)].

In Conditions A and B, the participants rated whether they liked the beer after tasting it only once. In Condition C, though, they made two ratings, first evaluating how much they liked the beer (X), and secondly rating how much they liked the sound-tasting experience (Y). The same analysis conducted as before was performed, but now considering Y ratings. Again, no significant main effect of condition was found, *F*_(2, 216)_ = 1.824, *p* = 0.164, η^2^_*p*_ = 0.017. Nevertheless, a significant effect was found when comparing before-after ratings, *F*_(1, 216)_ = 5.841, *p* = 0.016, η^2^_*p*_ = 0.026, and when assessing the interaction between before-after and condition, *F*_(2, 216)_ = 12.375, *p* < 0.001, η^2^_*p*_ = 0.103. As for the interaction term, in Condition C^10^, a significant difference was found between before and after-tasting ratings “Y” (*p* = 0.001), and between after-tasting ratings “X” and “Y” as well (*p* < 0.001). No differences were found between before-tasting and after-tasting ratings “X” (*p* > 0.999). Note that the means before and after-tasting “X” are 4.57 (*SD* = 1.12) and 4.91 (*SD* = 1.15), respectively. The mean of after-tasting “Y” was significantly higher (*M* = 5.53, *SD* = 1.41), when compared to each of the before and after-tasting “X” ones. The means of the three ratings corresponding to Condition C are shown in Figure 5.

### Visual and auditory effects on the perception of the beer

#### Taste ratings

**Figure 6:**
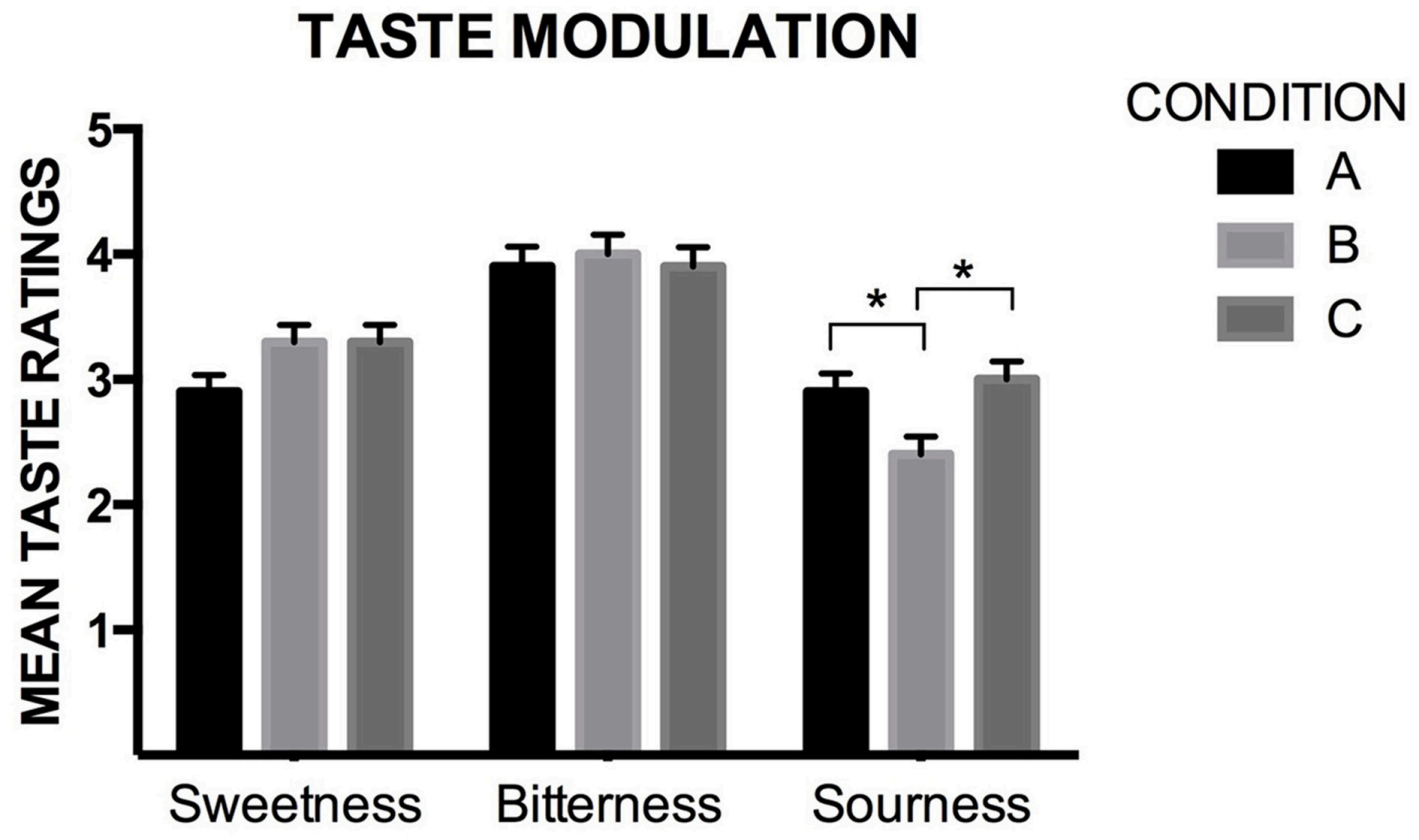
**Mean ratings of the levels of sweetness, bitterness, and sourness (based on 7-point rating scales, being 1 “not at all,” and 7 “Very much”)**. When observing the perceived levels of sourness, it can be seen that the participants rated the beer as significantly sourer in Condition A, than in Condition B. The same happened when comparing participants' ratings of Condition C with Condition B. Note that the levels of sourness in Conditions A and C are similar. It could be possible that, in Condition B, the design of the label neutralized the perceived sourness, and, in Condition C, the song may have enhanced it back again (significant differences between the specific interactions are indicated with an asterisk “*,” with *p*_*AB*_ = 0.023, and *p*_*BC*_ = 0.007).

In order to assess any potential modulatory effect of the condition (i.e., the beer's color, labeling, and sonic stimulus) on the sensory evaluation of the beer, a 3 × 3 mixed ANOVA was conducted with condition (A, B, and C) as the between-participants factor, and taste (sweetness, bitterness, and sourness) as the within-participants factor. No significant differences were observed between conditions, *F*_(2, 218)_ = 1.780 *p* = 0.171, η^2^_*p*_ = 0.016. A significant effect was found for taste ratings, *F*_(1.898, 413.746)_ = 52.072, *p* < 0.001, η^2^_*p*_ = 0.193 (note that, here, the Greenhouse–Geisser correction was used). The interaction between taste and condition was also significant, *F*_(4, 436)_ = 2.996, *p* = 0.019, η^2^_*p*_ = 0.027. Pairwise comparisons revealed that the bitter (*M* = 3.99, *SD* = 1.37) ratings were significantly higher than the sweet (*M* = 3.17, *SD* = 1.17) and sour (*M* = 2.77, *SD* = 1.28) ratings, and that the sweet ratings were significantly higher than the sour ratings (*p* ≤ 0.001, for all comparisons). As expected, since the taste of beer was being assessed, bitterness ratings were significantly higher than ratings of sweetness or sourness. As for the interaction term, the participants rated the beer as tasting significantly sourer in Conditions A and C, than in Condition B (*p*_*AB*_ = 0.023, and *p*_*BC*_ = 0.007, see Figure 6).

#### Perceived alcohol content (strength)

**Figure 7:**
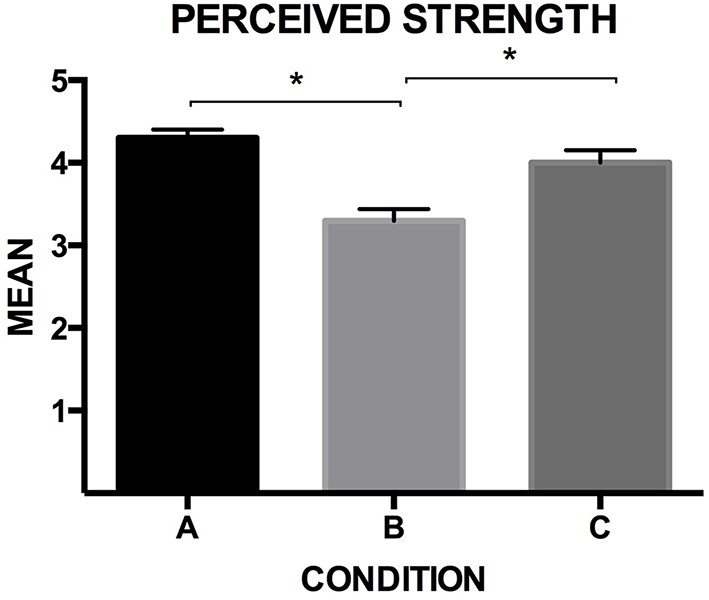
Means ratings and *SE* bars related to the beer alcoholic strength, in all cases (7-point rating scales, being 1 “not at all,” and 7 “Very much;” significant differences are indicated with an asterisk “*,” with *p* < 0.001, for both comparisons).

The participants rated the perceived alcohol content of the beer, in all conditions (see Figure 7). The effect of condition was significant, *F*_(2, 132)_ = 13.369, *p* < 0.001, η^2^_*p*_ = 0.168. The participants rated the beer as significantly stronger in Condition A than in Condition B, and significantly stronger in Condition C than in Condition B (*p* < 0.001, for both comparisons; *M*_*A*_ = 4.31, *SD* = 0.84, *M*_*B*_ = 3.37, *SD* = 1.23, *M*_*C*_ = 4.24, *SD* = 1.28). Here, it is interesting to note that, in Conditions B and C, the alcohol content of the beer was explicit in the label and thus, the results should be approached with some degree of caution. These results can be taken to suggest that the beer's dark color may have resulted in the participants rating it as stronger, when compared to its actual alcohol content (Spence et al., 2015).

### Willingness-to-pay

In all conditions, the participants had to rate—in euros—how much would they have been willing to pay for this beer, both, before and after having tasted it. A 3 × 2 mixed design ANOVA, with condition (A, B, and C) as the between-participant factor, and time (before vs. after tasting) as the within-participants factor was performed. Significant differences were found between conditions, *F*_(2, 217)_ = 10.756 *p* < 0.001, η^2^_*p*_ = 0.090. When comparing how much they would be willing to pay before and after tasting, no significant difference was found, *F*_(1, 217)_ = 0.157, *p* = 0.692, η^2^_*p*_ = 0.001. Further, the interaction between before-after-tasting and condition was not significant, *F*_(2, 217)_ = 0.162, *p* = 0.851, η^2^_*p*_ = 0.001. Pairwise comparisons revealed significant differences between Conditions A, and both B and C (AB *p* = 0.002, AC *p* < 0.001). From these results, it can be concluded that the participants were willing to pay significantly more when they were presented with the labeled beer bottle (*M*_*B*_ = 3.21, SD = 0.39) and the labeled beer bottle with the song together (*M*_*C*_ = 3.30, *SD* = 0.53), than when they were presented with the unlabeled beer (*M*_*A*_ = 2.93, *SD* = 0.76).

### Participants' knowledge of the editors and music/music-beer match ratings

#### Participants' knowledge of the editors

The participants in Conditions B and C reported their previous knowledge about TE. In both conditions, 37% reported knowing TE (*N* = 29 in both conditions). In order to understand how this previous knowledge may have influenced the before-and-after tasting results, the data were divided into two groups, one including the participants who reported knowing TE and the other, those that did not. ANOVAs were conducted for both liking and willingness to pay, with time (before vs. after tasting) as the within factor, and condition (B and C) as the between factor. Only a significant interaction between time and condition was found for the liking ratings of those participants who knew TE^11^, *F*_(1, 56)_ = 7.988 *p* = 0.007, η^2^_*p*_ = 0.125. In particular, these participants liked the beer significantly more after tasting it, in Condition C (*M*_before tasting C_ = 4.38, *SD* = 1.24; *M*_after tasting C_ = 5.00, *SD* = 1.21). On the other hand, in Condition B, the participants reported liking the beer less after tasting it (*M*_before tasting B_ = 4.86, *SD* = 1.22; *M*_after tasting B_ = 4.55 *SD* = 15.3).

#### Music and music-beer match ratings

The participants in Condition C also had to evaluate the song and how much they thought it matched with the beer's taste. Ratings show that most participants liked the song and most of them also agreed that it matched the beer's taste (Table 1).

## Discussion

### Summary of results

In this experiment, different groups of customers tasted a beer under three different testing conditions. Each condition was conceived in order to better understand how visual and auditory information, as captured in a beer tasting experience, can be used to add value to the experience of drinking beer. The first group tasted the beer after seeing a bottle without a label. The second group tasted the same beer, this time, after seeing the labeled bottle. Finally, the third group tasted this beer after seeing the labeled bottle, and while listening to a song, that was putatively congruent with the beer's profile. In part, these results provide original evidence for the idea that customized visual and auditory information can add value to the process of food and beverage product development, not to mention the subsequent enjoyment of those who eat and/or drink.

### Hedonic ratings and willingness-to-pay

The participants rated having liked the sound-beer tasting experience more when their attention was drawn toward both, the beer and the music, as a single multisensory experience (see Figure 5). By focusing on the music that was being played, people's attention was potentially drawn toward specific components of their sensory experience—in this case, toward the complexity of a craft-beer's taste (Driver, 2001; see Stevenson, 2012, for a review on the role of attention in flavor perception). The idea in this study was that the song could provide a complimentary effect, summed to the effect of the beer's label. As such, the participants were warned about the existence of a relationship between the beer and the song, and this could have drawn their attention to some key elements of the beer, such as its taste and/or strength (Spence, 2014).

The fact that most of the participants liked the song and agreed that it matched the taste of the beer (see Table 1) led them to like the beer/music combination more, when compared to their enjoinment while focusing on the beer's taste alone. That being said, it seems that people tend to like the sound-beer experience more when there is a clear—and positive—interaction between sound and taste. These results may be related to the concept of sensation transference (Cheskin, 1972). Similarly, Kantono et al. (2016) recently reported that listening to music can influence hedonic and sensory perception of food. They hypothesized that the overall hedonic judgment of the food (in this case, ice cream) was partially influenced by the hedonic valence of the music, and also by the hedonic tone of the ice cream itself. From a design perspective, future creators of similar food-music experiences might well want to take into account the suggestion that a positive hedonic evaluation of the sonic stimuli, and positive matching of the stimuli involved, may help people to better appreciate the overall multisensory tasting experience.

Importantly, those participants who knew The Editors and listened to the song (Condition C) reported having liked the beer more after tasting it, as compared to their pre-tasting ratings. In contrast, the participants that knew the band, but who only saw the beer's label (Condition B), reported having the opposite effect on their hedonic ratings (see Section “Participants' Knowledge of The Editors and Music/Music-Beer Match Ratings”). These results suggest that music may be effectively used to add value to multisensory tasting experiences when there is a previous connection between the participants and the music. Note that in this case, the music seems to have balanced a potential negative effect that the label might have induced in the overall experience. Other potential interactions between the label and the song are discussed in Section “Audiovisual Influences on the Perception of Beer”.

In the present study, the participants did not report being willing to pay more for a beer that came with its own song, as compared to the beer that came with a label. Although, the participants reported that they would have been willing to pay significantly more for this beer when presented with label and/or song (Conditions B/C), when compared to it without labeling (Condition A). These results contrast with those of a previous study, where people reported being willing to pay significantly more for a chocolate that came with its own customized soundscape (cf. Reinoso Carvalho et al., 2015b). Something important to remark here—and consider in future similar assessments—is that music is usually bounded to personal preferences and, hence, different songs can presumably lead to different emotional reactions.

### Audiovisual influences on the perception of beer

Concerning taste ratings (Section Visual and Auditory Effects on the Perception of the Beer), the song seemed to have a modulatory effect on the perceived sourness of the beer. This result is compatible with the musical and psychoacoustic analysis of the sonic stimulus. However, the ratings of Conditions A and C are mostly indistinguishable, and significantly higher when compared to the ratings in Condition B. Similarly, the participants reported that the beer tasted significantly stronger when it was presented without labeling (Condition A), and in Condition C, when the beer's presentation was accompanied by the song (see Section Visual and Auditory Effects on the Perception of the Beer for results), than in Condition B. In the two cases mentioned above, it would seem that drawing attention to the visual aspects of the label, in Condition B, had a negative effect. In particular, we suggest that in Condition B, the semantic contents of the label may have counterbalanced the perceived sourness, and, in Condition C, the song may have enhanced it (see Section “Visual and Auditory Effects on the Perception of the Beer”). Another potential relevant factor present in the label was the visual impact of the diagonal white line (see Figure 1). Such line goes from top left down to bottom right. Youssef et al. (2015) recently reported that, potentially, consumers have a preference for an oblique line ascending to the right, when evaluating plating arrangements. Something similar is likely to be found with product packaging. In summary, the white line was in the opposite direction as the probable preferred choice of the customers that experienced the label.

### Limitations and future work

This experiment was implemented in a brewery with its own customers and, hence, all of the participants were constantly influenced by the brand (for instance, think of the fact that all participants tasted the beer using the brewer's own glassware; see Figure 2), which potentially provided brand-specific cues that may also have contributed to the findings. Future research could develop a similar experience in a more typical drinking environment, such as a common bar, including neutral glassware. A more balanced audience would also be useful to assess the influence of brand familiarity (see footnote 2).

As previously explained, all of the participants used headphones, including the ones that didn't listen to the song, and this may have reduced the ecological value of the set-up. The outcome of such a setting might have been less enjoyment for the participants who did not listen to the song, and this could have affected their overall hedonic assessment. Important to note though, is the fact that headphones are already included as part of commercial dinning settings. For instance, The Fat Duck Restaurant (UK) offers to its clients a dish called “Sound of the Sea.” Part of its presentation includes a sound reproduction system accompanied by a pair of ear buds (Spence and Piqueras-Fiszman, 2014). Yet, future studies could rehearse the usage of state-of-the-art immersive soundscaping systems (such as Ambisonics, wave field synthesis), in order to provide the same sonic information to all participants, at all times, regardless the existent background noise conditions^12^.

When discussing the hedonic ratings presented here, attentional redirection is suggested as one of the mechanisms that prompted the observed enjoyment (Spence, 2014). People's attention may be drawn to customized/congruent sonic cues, in order to observe an effect on the enjoyment of the drink, as attentional effect may be enhanced for familiar stimuli (Spence and Wang, 2015). Future studies could assess how adding sound as part of tasting experiences may generally affect hedonic ratings related to food/beverage consumption, despite the fact that such sound might be—or not—congruent with the food/beverage being tasted (Reinoso Carvalho et al., in press). For instance, two new control conditions may be added to the existing experimental design for further understanding of the effects of music on the perception of the beer's taste. Using this study as template, think, for example, of a new control group of participants that drinks the “Salvation” beer while listening to another song, and another control group that drinks a different beer while listening to “Oceans of Light.”

The ratings of the beer's strength were significantly different when comparing Conditions A/C to Condition B. We believe that the dark color of the beer may have caused such strength modulation. Although, here it is important to note that most of the participants were Belgian (or from its surroundings). In this European region, dark ale beers are usually related to higher alcohol levels^13^. It would be interesting, in the future, to develop an experience specifically focused on the potential perceptual modulatory effects of a beer's color on its alcoholic strength and, this time, considering cultural variability of the sample (Wan et al., 2014)^14^.

In the present study, it was not possible to discriminate the influence of the given messages in Conditions B and C^15^ (cf. Reinoso Carvalho et al., 2015b). A future implementation could consider delivering such a message only to the participants being stimulated by a song (i.e., in this experiment, only to the participants in Condition C). This way, it could be possible to eliminate any triggering effects of possible musical associations to participants that could be, for example, familiar with the band, but not listening to the song.

It is feasible to effectively include emotional cues, cultural, and social contexts, or even pair psychoacoustic and musical elements as part of a beer's formula. Future related work could improve this approach by assessing, for instance, the “melody of the beer” by means of temporal dominance of sensations (TDS), and/or temporal dominance of emotions (TDE). Such measurements are usually based on intensity, order, and speed of the successive dominant flavor aspects of food/beverages (e.g., Jager et al., 2013, 2014). For the brewery industry, the consideration of these methods while evaluating sound-taste interactions could bring results that might be easier to include as part of their workflow.

Multisensory beer design can potentially provide beneficial or adverse effects in terms of decreasing/increasing alcohol consumption. In this experiment, a fixed quantity of beer was served to all participants, meaning that there was no measurement of beer amount consumption. A future experience could, for example, hypothesize as to how a congruent vs. incongruent sonic stimulus may affect the physiological consumption of beer (i.e., speed and/or amount).

### Final remarks

The creative process involved in our work could be of value while conceiving, for instance, food/beverage packaging that includes sound. Furthermore, all of this brainstorming may be used with the objective of creating a stronger beer profile, and/or eventually balancing (the perception of) its formula. It is also worth mentioning that the younger generations (e.g., Millennials) are more and more interested in experiences that are able to enhance their sensory experiences (Leow, 2015) that they offer. Hence, food/drink experiences involving Sensploration techniques seem to already have a steady—and growing—audience. A more artistic approach in food/beverage product development may end up bringing more scientific and technological inspiration into common aspects of food design, and vice-versa.

## Author contributions

All authors contributed in the entire process of developing this report. From the experimental design, passing through the development and characterization of stimuli, sampling data, processing, and analysing results.

## Funding

This research was supported by the Rethinking the Senses grant from the AHRC (UK) awarded to Charles Spence (AH/L007053/1). FR was partly funded by the CAPES Foundation, Brazil (BEX 3488/13-6). RV was supported by the Flemish Methusalem program (METH/14/02 to J. Wagemans), the EU Horizon 2020 program (HealthPac to J. van Opstal), and the Flemish Organization for Scientific Research (FWO).

### Conflict of interest statement

The authors declare that the research was conducted in the absence of any commercial or financial relationships that could be construed as a potential conflict of interest.

## Footnotes

^1^Retrieved from http://www.dailymail.co.uk/news/article-3168913/If-meal-looks-good-think-tastes-better-Simply-arranging-food-carefully-plate-persuade-diners-pay-three-times-it.html (July, 2015).

^2^Eighty-three percent of the participants reported knowing TBP (*n* = 191). When asked how often the participants consumed products from TBP—on a 7 point scale, with 1 corresponding to “never” and 7 to “very often”—the mean of their answers was 3.30 (*SD* = 1.80). Note that, since the vast majority of the participants reported knowing TBP, in this study it was not possible to include in our data analysis control for familiarity with the beer's brand.

^3^See http://www.editorsofficial.com/ (retrieved November 2015).

^4^See http://2.bp.blogspot.com/-7f0GBxYcIvU/Tkn9tmAx_HI/AAAAAAAAABQ/UjnepOkh7-Q/s1600/Picture+9.png for an EBC classification (Retrieved December, 2015).

^5^The International Bittering Units scale (IBU) is a standardized quantitative value designated to the measurement of bitterness of the beer.

^6^They claim that the font has a “strong-unique personality, without being aggressive.”

^7^Retrieved from http://streamd.hitparade.ch/cdimages/editors-in_dream_a.jpg (November 2015).

^8^See http://www.beerproject.be/en/beers for other examples of TBP's labels (retrieved December 2015).

^9^Link to the song—https://play.spotify.com/track/4yVv19QPf9WmaAmYWOrdfr?play=true&utm_source=open.spotify.com&utm_medium=open(retrieved January 2016).

^10^Before-tasting, after-tasting ratings “X,” and after-tasting ratings “Y.”

^11^Here, there were no significant differences for the ratings of willingness-to-pay of the participants who knew TE [*F*_(1, 56)_ = 0.242 *p* = 0.624, η^2^_*p*_ = 0.004].

^12^Interesting also to mention here that, at the moment, spatial, and 3D audio solutions are getting special attention due to the high impact of VR headsets in the market. That being said, it is plausible to assume that, in the near future, headsets will most likely be considered as an essential component in virtual studies.

^13^http://www.beeradvocate.com/beer/style/119/ (retrieved December, 2015).

^14^Previous studies have agreed that the color of the beer has an influence on general assessments while analyzing and comparing them (Guinard et al., 1998, 2000, 2001). For example, Guinard et al. (2000) reported that young men's hedonic beer evaluations tended to track the drink's color.

^15^*Message in Condition B:* this beer results from a collaboration between The Beer Project and The Editors. *Message in Condition C*: this song was the source of inspiration for this formulation of this beer.

## Appendix A: the participant's evaluation of the beer's labeling

During this beer tasting experience, the participants who viewed the label (Condition B) and label + song (Condition C), rated a few basic aspects of the visual presentation of the bottle. Table A1 shows the questions that were asked, and the corresponding ratings.

**Table A1:** **These ratings show us that most of the participants liked the label and its colors**.

**Question**	***Mean***	***SD***
1. How much do you like the bottle's label?	4.73	1.42
2. How much do you like the colors of the bottle's label?	4.55	1.44
3. Evaluate the round/angular shaping of the typography of the bottle's label	3.31	1.12

*When compared on an angular/round scale—with 1 being “very angular,” 4 “balanced,” and 7 “very round”—, the typography was mostly evaluated as balanced*.
